# Epigenetic repression of PDZ-LIM domain-containing protein 2 promotes ovarian cancer via NOS2-derived nitric oxide signaling

**DOI:** 10.18632/oncotarget.6368

**Published:** 2015-11-22

**Authors:** Linjie Zhao, Chuan Yu, Shengtao Zhou, Wayne Bond Lau, Bonnie Lau, Zhongyue Luo, Qiao Lin, Huiliang Yang, Yu Xuan, Tao Yi, Xia Zhao, Yuquan Wei

**Affiliations:** ^1^ Department of Gynecology and Obstetrics, Key Laboratory of Obstetrics & Gynecologic and Pediatric Diseases and Birth Defects of Ministry of Education, West China Second Hospital, Chengdu, China; ^2^ The State Key Laboratory of Biotherapy, West China Hospital, Sichuan University, Chengdu, China; ^3^ Department of Emergency Medicine, Thomas Jefferson University Hospital, Philadelphia, PA, USA; ^4^ Department of Surgery, Emergency Medicine, Kaiser Permanente Santa Clara Medical Center, Santa Clara, CA, USA; ^5^ College of Biological Sciences, Sichuan University, Chengdu, China; ^6^ Department of Orthopedics, West China Hospital, Sichuan University, Chengdu, China

**Keywords:** ovarian cancer, PDLIM2, DNA methylation, NOS2, nitric oxide signaling

## Abstract

Ovarian cancer constitutes one of the most lethal gynaecological malignancies worldwide and currently no satisfactory therapeutic approaches have been established. Therefore, elucidation of molecular mechanisms to develop targeted therapy of ovarian cancer is crucial. PDLIM2 is critical to promote ubiquitination of nuclear p65 and thus its role in inflammation has been highlighted recently. We demonstrate that PDLIM2 is decreased in both ovarian high-grade serous carcinoma and in various human ovarian cancer cell lines compared with normal ovary tissues and human ovarian surface epithelial cells (HOSE). Further functional analysis revealed that PDLIM2 is epigenetically repressed in ovarian cancer development and inhibition of PDLIM2 promoted ovarian cancer growth both *in vivo* and *in vitro via* NOS2-derived nitric oxide signaling, leading to recruitment of M2 type macrophages. These results suggest that PDLIM2 might be involved in ovarian cancer pathogenesis, which could serve as a promising therapeutic target for ovarian cancer patients.

## INTRODUCTION

Ovarian cancer occurs in 1 of 2500 post-menopausal females in the United States and has been considered the most lethal gynecologic malignancy, accounting for 5–6% of all cancer-related mortalities worldwide [1]. Patients with advanced ovarian cancer are mainly treated with surgical cytoreduction, followed by chemotherapy, with a clinical response rate of 60–70%. However, many patients ultimately succumb to relapse resistant to chemotherapy, yielding an overall 5-year 10-30% survival rate [2]. Therefore, it is imperative to develop novel therapeutic strategies treating advanced or recurrent ovarian cancer.

The role of inflammation in ovarian carcinogenesis was first proposed in the ‘incessant ovulation theory’ [3]. Rupture of the ovarian surface epithelium induces an inflammatory reaction causing cell damage and proliferation, enhancing potential aberrant DNA repair, inactivating tumor-suppressor genes, leading to subsequent mutagenesis. Chronic diseases of the female reproductive tract (including endometriosis, polycystic ovary syndrome and pelvic inflammatory disease) are associated with inflammation, and are purported epithelial invasive ovarian cancer (EOC) risk factors [4]. Recently, both mouse and human studies suggest nuclear factor-κB (NF-κB) transcription factor plays a causative role in inflammation, and subsequently, the pathogeneses of ovarian cancer [5]. Deregulation of NF-κB activity may influence the outcome in women receiving standard therapy for advanced ovarian cancer. Moreover, as a key regulator of NF-κB signaling, inhibitors of IkB kinases (IKK) were recently demonstrated to play a critical role in both invasive and metastastic ovarian cancer processes [6]. These observations demonstrate NF-κB signaling is vital in ovarian cancer oncogenesis and progression. NF-κB activity is tightly regulated during physiologic conditions. Typically, NF-κB is rapidly activated transiently in response to different stimuli. One essential mechanism for the swift termination of the NF-κB response involves nuclear degradation of its prototypic member p65, a process predominantly controlled by PDZ-LIM domain-containing Protein 2 (PDLIM2) [7]. PDLIM2 is the most recently discovered PDZ-LIM domain-containing protein. The COOH-terminal LIM domain of PDLIM2 is essential for promoting ubiquitination of nuclear p65, whereas its NH2-terminal PDZ domain participates in shuttling nuclear p65 along the nuclear framework into discrete intranuclear compartments for proteasome-mediated degradation. Accordingly, PDLIM2 knockout mice are more sensitive to lipopolysaccharide(LPS)-induced shock due to enhanced NF-κB/p65 activation and augmented inflammatory cytokines production [8].

Currently, the specific molecular mechanisms underlying ovarian cancer pathogenesis remains unclear. Herein, we demonstrate PDLIM2 is decreased in both ovarian high-grade serous carcinoma and in various human ovarian cancer cell lines compared to normal ovarian tissues and human ovarian surface epithelial cells (HOSE). Further functional analysis revealed PDLIM2 is epigenetically repressed in ovarian cancer development. Inhibition of PDLIM2 promoted ovarian cancer growth both *in vivo* and *in vitro* via NOS2-derived nitric oxide signaling, leading to M2 type macrophages recruitment. These results suggest an important role of PDLIM2 in ovarian cancer pathogenesis, which might be a promising therapeutic target in the clinical treatment of ovarian cancer.

## RESULTS

### PDLIM2 expression is repressed in ovarian cancer and is associated with patient prognosis

**Figure 1:**
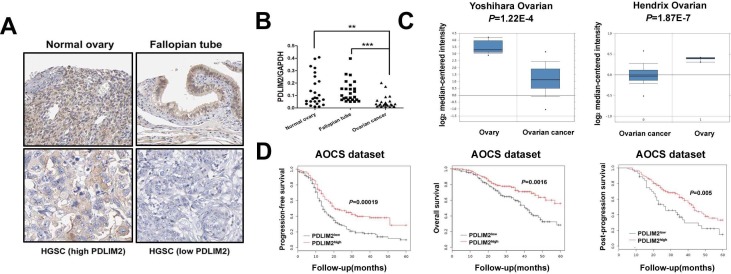
Repression of PDLIM2 is associated with ovarian cancer prognosis **A.** immunolocalization of PDLIM2 showing high PDLIM2 protein expression in normal ovarian and fallopian tube tissue samples and low PDLIM2 expression in high-grade serous ovarian carcinoma samples. HGSC: high-grade serous ovarian carcinoma. **B.** scatter plot showing the distribution of relative PDLIM2 expression intensity in the normal ovarian tissues, normal fallopian tube tissues, and high-grade serous ovarian carcinoma tissues. **C.** box plot showing significantly lower PDLIM2 protein expression in ovarian tumor samples compared with normal ovarian tissues in both Yoshihara dataset and Hendrix dataset. **D.** Kaplan–Meier analysis of the Australian Ovarian Cancer Study(AOCS) patients with ovarian carcinoma showing a significant correlation between PDLIM2 protein expression and progression-free survival, overall survival, and post-progression survival. *, *P* < 0.05; **, *P* < 0.01; ***, *P* < 0.001.

**Figure 2:**
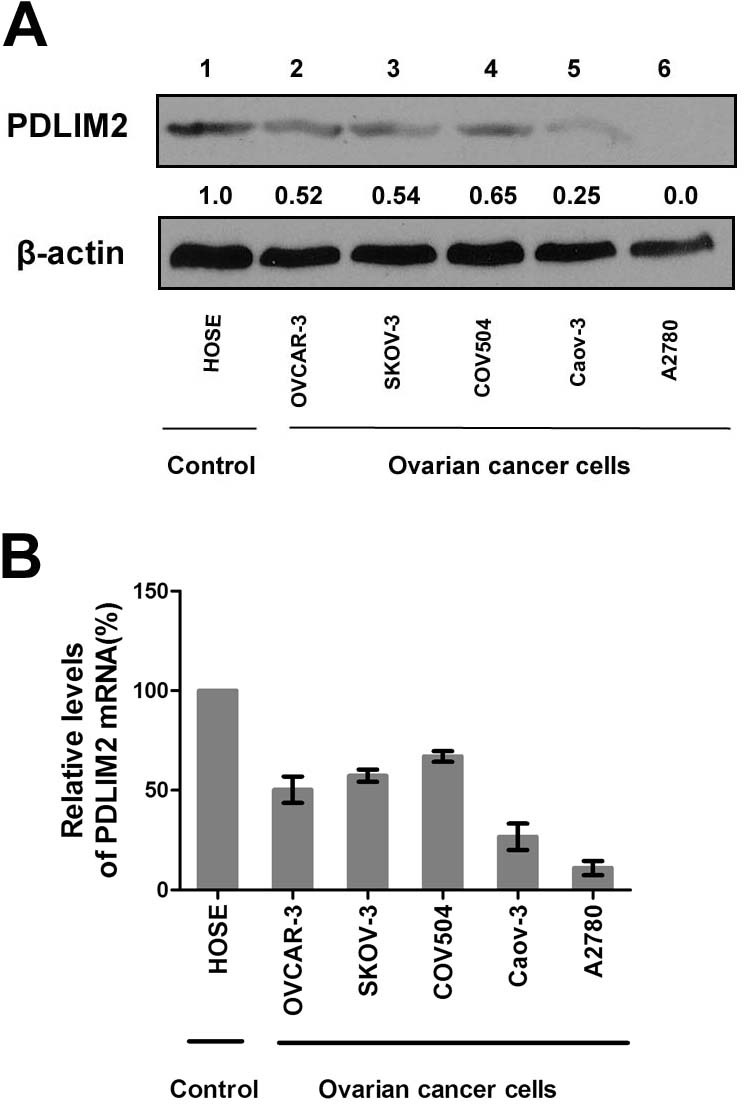
Repression of PDLIM2 expression in ovarian cancer cell lines **A.** Protein expressions of PDLIM2 in the indicated ovarian cancer cell lines and human ovarian surface epithelial cells(HOSE). β-actin was used as a loading control. **B.** The relative levels of PDLIM2 mRNAs in the indicated ovarian cancer cells and HOSE cells were analyzed by real-time PCR using GAPDH mRNA as a control and represented as percentile of that in HOSE cells (set as 100). The data presented are the mean±S.D. (error bars).

Given the critical role of NF-κB in ovarian cancer pathogenesis and the involvement of PDLIM2 in terminating NF-κB activation, we hypothesized PDLIM2 is involved in the ovarian cancer pathogenesis. We determined PDLIM2 levels in normal ovary, fallopian tube, and high grade serous ovarian carcinoma (HGSC) specimens. PDLIM2 protein expression is significantly decreased in HGSC specimens (Figure 1A). Further RT-PCR analysis revealed PDLIM2 mRNA level is significantly decreased in ovarian cancer tissues compared to normal ovary and fallopian tube tissues (Figure 1B). We next investigated previously published microarray data regarding PDLIM2 expression in the Oncomine database. PDLIM2 mRNA levels were decreased in ovarian cancer tissues compared to normal tissue in the Yoshihara and Hendrix datasets (*P*=1.22E-4 and *P*=1.87E-7, respectively) (Figure 1C). We next performed quantitative real-time PCR and immunoblotting to respectively determine PDLIM2 mRNA and protein levels in ovarian cancer cells and control HOSE cells. Both PDLIM2 mRNA levels and protein levels are significantly decreased in all ovarian cancer cell lines compared to control (Figure 2A and 2B). Survival analysis revealed PDLIM2 repression was associated with decreased progression-free survival, overall survival, and post-progression survival (Figure 1D). These data suggest PDLIM2 protein expression is repressed in ovarian cancer, and its decreased expression is associated with poor prognosis in ovarian cancer patients.

### PDLIM2 expression attenuates ovarian cancer growth

**Figure 3:**
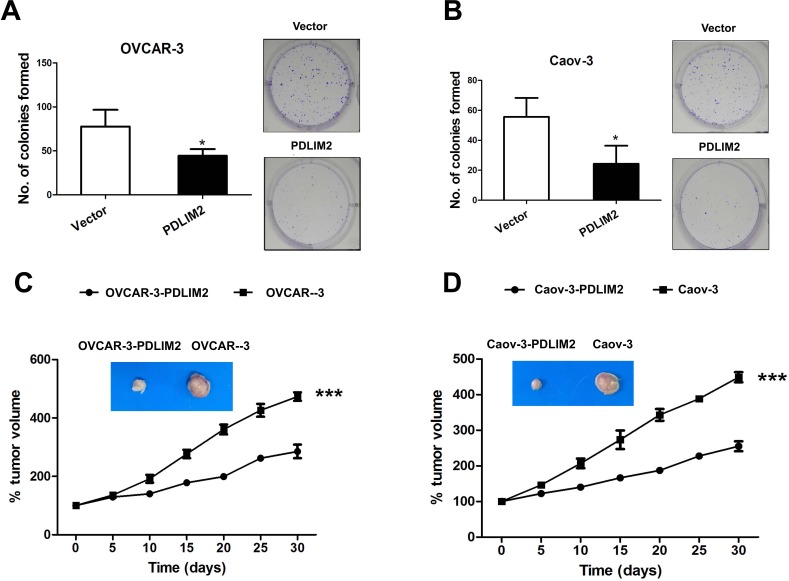
Suppression of tumorigenicities of ovarian cancer cells by PDLIM2 **A.** colony formation analysis of human OVCAR-3 ovarian cancer cells transfected with PDLIM2 compared with control. **B.** colony formation analysis of human Caov-3 ovarian cancer cells transfected with PDLIM2 compared with control. **C.** growth suppression of OVCAR-3 ovarian cancer cancer cells by PDLIM2. **D.** growth suppression of Caov-3 ovarian cancer cancer cells by PDLIM2. The data presented are the mean±S.D. (error bars). *, *P* < 0.05; **, *P* < 0.01; ***, *P* < 0.001.

To further investigate the effect of PDLIM2 expression in ovarian cancer, we performed a colony formation assay, employing OVCAR-3 and Caov-3 cells expressing PDLIM2 versus empty vector. Both OVCAR-3 cells (Figure 3A) and Caov-3 cells (Figure 3B) expressing PDLIM2 formed markedly fewer and smaller colonies compared to control, suggesting PDLIM2 suppresses ovarian cancer growth *in vitro*. To validate these observations *in vivo*, we separately incubated the control vector and PDLIM2-expressing ovarian cancer cells subcutaneously in nude mice. As shown in Figure 3C and 3D, both control vector and PDLIM2-expressing cells of OVCAR-3 and Caov-3 cells developed tumors subcutaneously in nude mice. However, PDLIM2-expressing ovarian cancer cells were significantly smaller than those formed by the vector-expressing cell lines. Taken together, these data support PDLIM2-mediated suppression of ovarian cancer cell tumorigenicity.

### PDLIM2 repression in ovarian cancer involves DNA methylation

**Figure 4:**
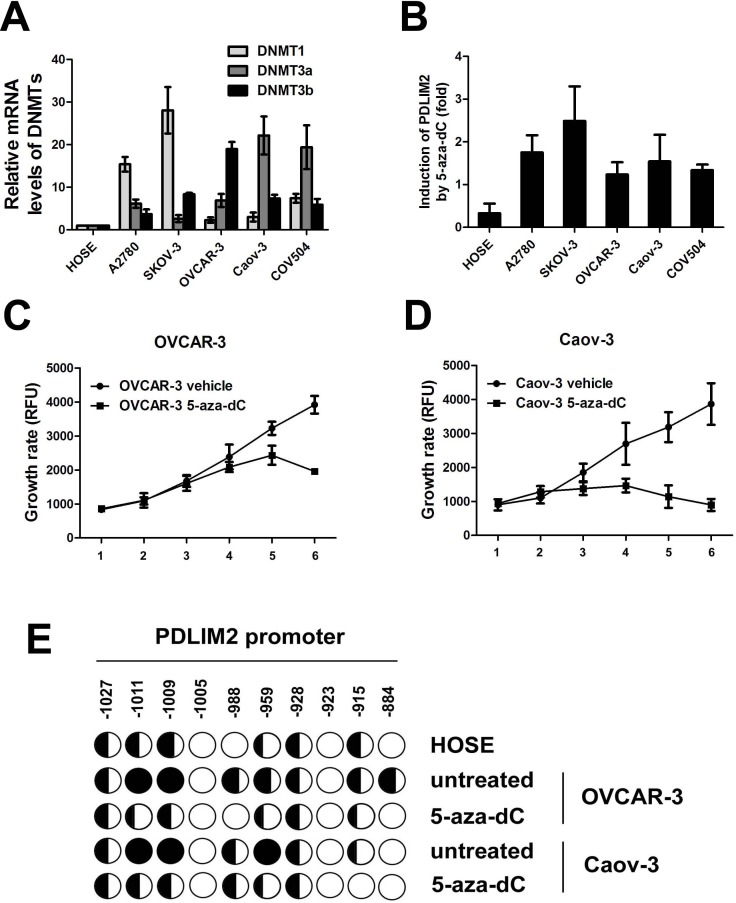
DNA methylation-mediated repression of PDLIM2 in ovarian cancer cells **A.** Relative mRNA levels of DNMT1, DNMT3a, and DNMT3b in human ovarian cancer cells and HOSE cells by RT-PCR analysis. **B.** 5-aza-dC-mediated recovery of PDLIM2 expression in human ovarian cancer cells and HOSE cells by RT-PCR analysis. PDLIM2 mRNA levels in the 5-aza-dC- or mock-treated cells were also analyzed by RT-PCR. The PDLIM2 inductions by 5-aza-dC are represented as percentile of that in mock-treated HOSE cells. **C.** growth curve of OVCAR-3 ovarian cancer cells treated by 5-aza-dC. **D.** growth curve of Caov-3 ovarian cancer cells treated by 5-aza-dC. **E.** methylation of the PDLIM2 promoter in ovarian cancer cells. The indicated cell lines were treated with 5 μM 5-aza-dC or vehicle for 5 days, followed by the bisulfite genomic DNA sequencing. Each circle represents a CpG site; open circles represent unmethylated CpG dinucleotides, and filled circles represent methylated CpG sites. The ratios of the filled area in circles represent percentiles of the methylation in the CpG sites. The position of each CpG nucleotide relative to the PDLIM2 transcription initiation site (+1) is indicated at the top. The data presented are the mean±S.D. (error bars)

Next, we investigated the underlying mechanism of PDLIM2 repression in ovarian cancer, and asssessed the potential role of DNA methylation, a major mechanism responsible for tumor suppressor gene suppression in malignant cells. We first analyzed the expression levels of three DNA methyltransferases responsible for DNA methylation (DNMT1, DNMT3a, and DNMT3b). All three enzymes were significantly increased in each ovarian cancer cell line compared to nontumorigenic HOSE cells (albeit to different degrees Figure 4A). To further establish a correlation between DNA methyltransferase and PDLIM2 expression, we determined the effect of 5-aza-dC, a highly specific DNA methyltransferase inhibitor, upon cancer cell PDLIM2 expression. 5-aza-dC treatment restored PDLIM2 expression in all studied ovarian cancer cell lines (Figure 4B). These data suggest PDLIM2 repression in ovarian cancer involves DNA methylation.

Interestingly, 5-aza-dC treatment also resulted in growth inhibition of OVCAR-3 and Caov-3 ovarian cancer cells (Figure 4C and 4D). To determine whether the PDLIM2 promoter is methylated in ovarian cancer cells, and whether 5-aza-dC–mediated restoration of PDLIM2 expression involves inhibition of PDLIM2 promoter methylation, we performed bisulfite genomic DNA sequencing. The PDLIM2 promoter was hypermethylated in ovarian cancer cell lines compared to HOSE cells (Figure 4E). 5-aza-dC administration significantly reduced methylation of the PDLIM2 promoter. Together, these data suggest promoter methylation is an important modulating factor in the expression of PDLIM2 in ovarian cancer.

### PDLIM2 inhibition increases endogenous NO level

**Figure 5:**
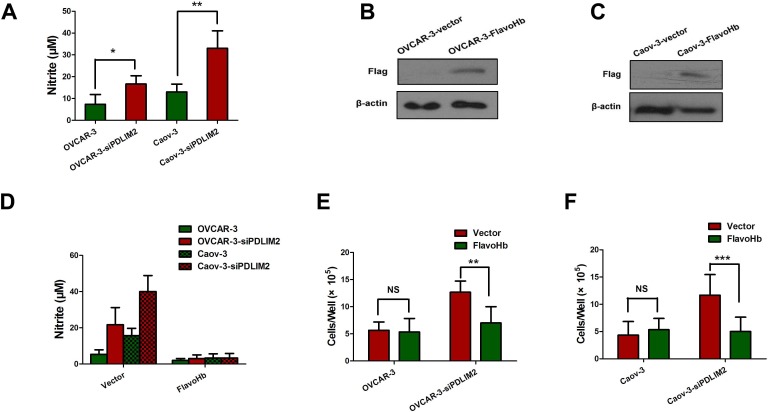
PDLIM2 repression triggers increased NO synthesis in ovarian cancer **A.** Nitrite (NO_2_-) was quantified in the media of different cells. **B.** immuobloting of flag-tagged FlavoHb expression in OVCAR-3 ovarian cancer cells. **C.** immuobloting of Flag-tagged FlavoHb expression in Caov-3 ovarian cancer cells. **D.** FlavoHb expression decreased the total nitrite measured in media in PDLIM2-repressed ovarian cancer cells. **E.** FlavoHb impairs growth of PDLIM2-repressed OVCAR-3 cancer cells. **F.** FlavoHb impairs growth of PDLIM2-repressed Caov-3 cancer cells. The data presented are the mean±S.D. (error bars). *, *P* < 0.05; **, *P* < 0.01; ***, *P* < 0.001.

We next investigated consequential downstream molecular events of PDLIM2 repression in ovarian cancer. We first compared the NO production capacity of PDLIM2-repressed OVCAR-3 and Caov-3 ovarian cancer cells by measuring nitrite (NO_2_-, a stable byproduct of NO) in culture medium. Both siPDLIM2-treated OVCAR-3 and Caov-3 ovarian cancer cells produced significantly more NO than control (Figure 5A), suggesting PDLIM2 repression may increase NO synthesis. To investigate the function of endogenous NO in ovarian cancer cells, we transfected OVCAR-3 and Caov-3 ovarian cancer cells with *E. coli* FlavoHb, a potent NO-consuming enzyme that converts NO to nitrate (NO_3_-) (Figure 5B and 5C). FlavoHb reduced NO synthesis in PDLIM2-repressed ovarian cancer cells (Figure 5D), and inhibited both OVCAR-3 and Caov-3 cellular growth (Figure 5E and 5F). Taken together, these data suggest PDLIM2 inhibition increases endogenous NO levels, with increased resultant ovarian cancer cell growth.

### NOS2 expression is increased in PDLIM2-repressed ovarian cancer cells

**Figure 6:**
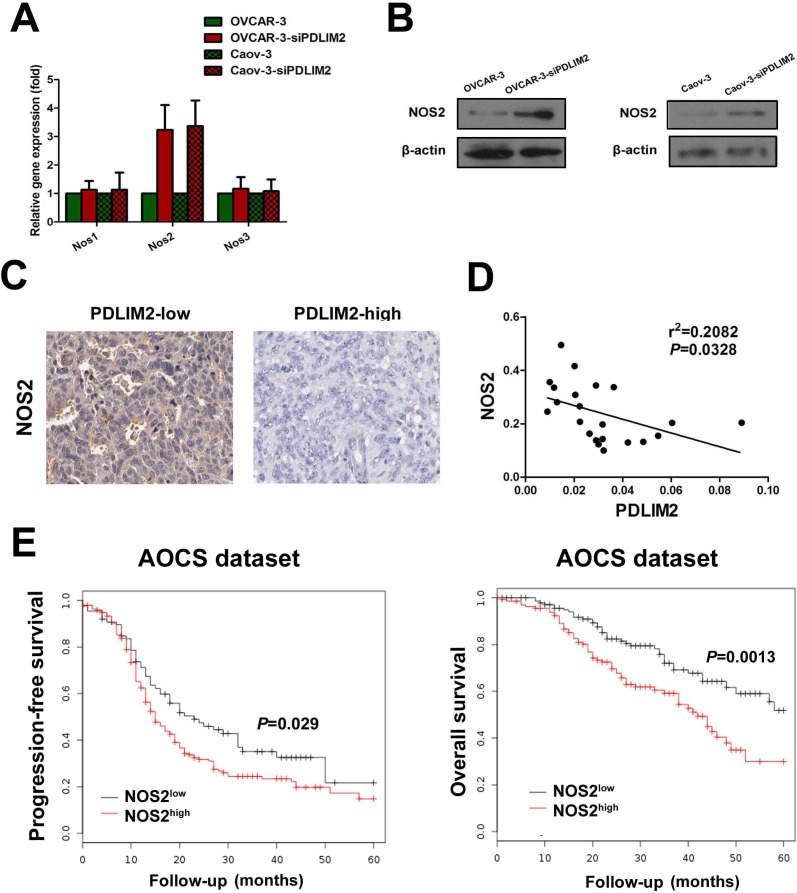
Enhanced expression of NOS2 in PDLIM2-repressed ovarian cancer cells **A.** detection of NOS1, NOS2, and NOS3 expression in PDLIM2-repressed OVCAR-3 and Caov-3 ovarian cancer cells and control by RT-PCR. **B.** detection of NOS1, NOS2, and NOS3 expression in PDLIM2-repressed OVCAR-3 and Caov-3 ovarian cancer cells and control by immunoblotting. **C.** NOS2 immunostaining of paraffin-embedded PDLIM2-high and PDLIM2-low ovarian cancer specimens. **D.** correlation of NOS2 and PDLIM2 expression in ovarian cancer specimens. **E.** Kaplan–Meier analysis of the Australian Ovarian Cancer Study(AOCS) patients with ovarian carcinoma showing a significant correlation between NOS2 protein expression and progression-free survival and overall survival.

Although we demonstrated FlavoHb blocked NO availability and decreased ovarian cancer cell growth, the source of PDLIM2-repressed cell-derived NO remains unclear. There are three isoforms of nitric oxide synthases: NOS1, NOS2 (iNOS), and NOS3 (eNOS). We determined the mRNA and expression levels of these three NOS isoforms to identify which is responsible for increased NO synthesis in PDLIM2-repressed ovarian cancer cells. Via RT-PCR analysis, both OVCAR-3 and Caov-3 ovarian cancer cells exhibit elevated NOS2 mRNA levels after siPDLIM2 treatment (Figure 6A), consistent with the finding confirmed by immunoblotting (Figure 6B). No significant change was observed in the NOS1 and NOS3 mRNA levels (Figure 6A). Immunohistochemistry analysis revealed NOS2 expression is significantly increased in PDLIM2-repressed ovarian cancer specimens (Figure 6C). Correlation analysis demonstrated tumor expression of NOS2 is negatively correlated with PDLIM2 levels (r^2^=0.2082; *P*=0.0328) in ovarian cancer specimens, suggesting a functional link between the two factors (Figure 6D). Additionally, survival analysis revealed increased expression of NOS2 was associated with decreased progression-free survival and overall survival (Figure 6E). These data demonstrate NOS2 may be an important mediator in PDLIM2-repression-induced NO synthesis in ovarian cancer.

### Inhibition of NO synthesis attenuates ovarian cancer growth *in vitro* and *in vivo*

**Figure 7:**
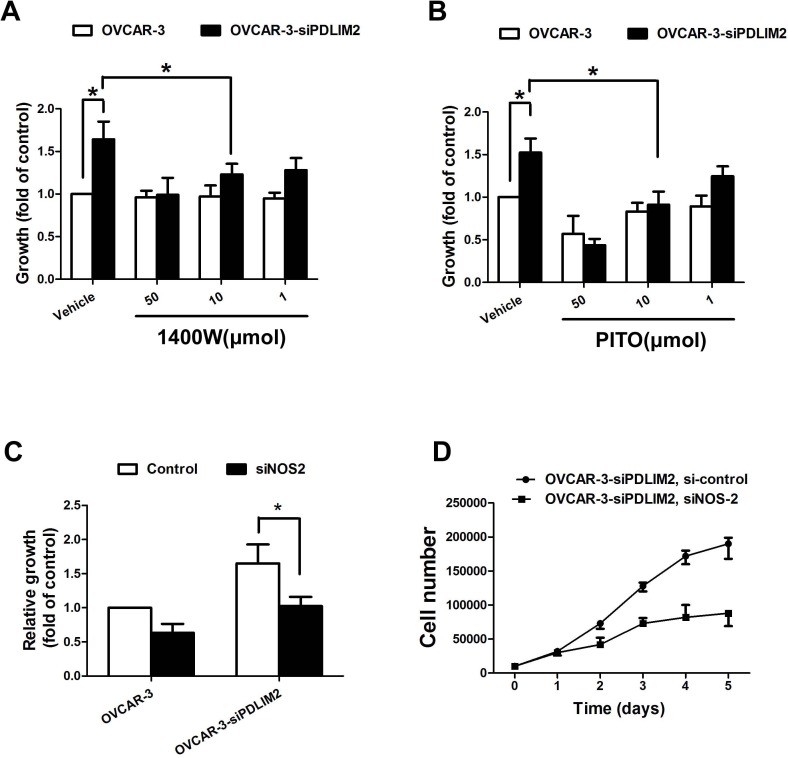
Impaired growth of PDLIM2-repressed ovarian cancer cells under NOS2/NO inhibition *in vitro* **A.** growth of PDLIM2-repressed OVCAR-3 cells and control after treatment with 1400W. **B.** growth of PDLIM2-repressed OVCAR-3 cells and control after treatment with PITO. **C.** growth of PDLIM2-repressed OVCAR-3 cells and control after treatment with NOS2 siRNA. **D.** grow curve of PDLIM2-repressed OVCAR-3 cells and control after treatment with NOS2 siRNA. The data presented are the mean±S.D. (error bars). *, *P* < 0.05; **, *P* < 0.01; ***, *P* < 0.001.

**Figure 8:**
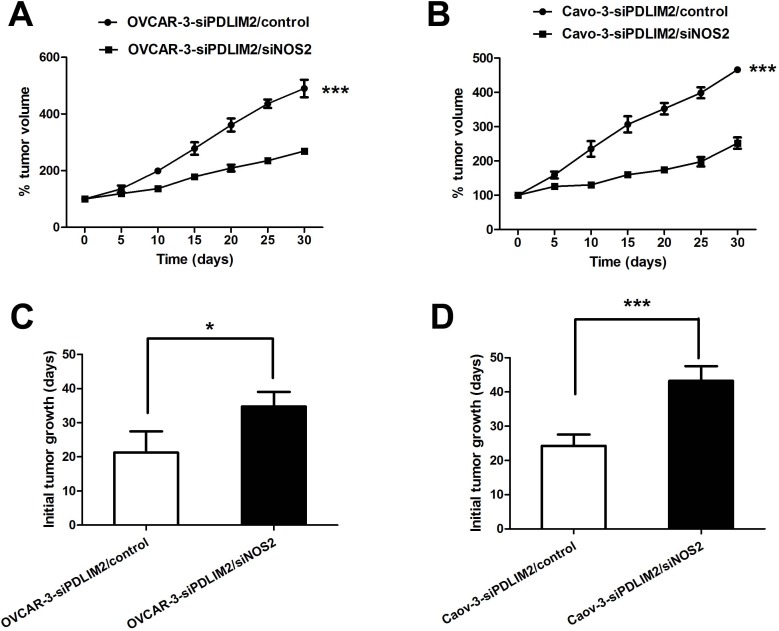
NOS2 inhibition reduces PDLIM2-repressed ovarian cancer growth *in vivo* **A.** growth curves of PDLIM2-repressed OVCAR-3 cells treated with siNOS2 or control *in vivo*. **B.** growth curves of PDLIM2-repressed Caov-3 cells treated with siNOS2 or control *in vivo*. **C.** length of time to reach 15 mm^3^ tumor size of PDLIM2-repressed OVCAR-3 cells treated with siNOS2 or control. **D.** length of time to reach 15 mm3 tumor size of PDLIM2-repressed Caov-3 cells treated with siNOS2 or control. The data presented are the mean±S.D. (error bars). *, *P* < 0.05; **, *P* < 0.01; ***, *P* < 0.001.

To further evaluate the functional import of NO in ovarian cancer growth, we assessed OVCAR-3 and Caov-3 cells *in vitro* during conditions of impaired NOS2/NO signaling. Both NOS2 inhibitor 1400W (Figure 7A) and PTIO (Figure 7B) significantly reduced the growth rate of siPDLIM2-treated ovarian cancer cells compared with control. To directly determine the importance of NOS2 in siPDLIM2-treated ovarian cell growth, we employed NOS2-targeting siRNA for functional validation. Impaired NOS2 expression reduced siPDLIM2-treated OVCAR-3 and Caov-3 cell growth *in vitro*, compared to control (Figure 7C and 7D). To examine the role of NO synthesis in ovarian cancer growth *in vivo*, we treated PDLIM-2-repressed OVCAR-3 and Caov-3 cancer cells with NOS2 siRNA or control. NOS2-siRNA decreased both OVCAR-3 (Figure 8A) and Caov-3 (Figure 8B) cancer cell growth *in vivo*, in statistically significant fashion (Figure 8C and 8D). These data support the implications of NOS2-induced NO synthesis upon ovarian cancer cell growth.

### PDLIM2 repression recruits M2 type tumor-associated macrophage infiltration in ovarian cancer, reversed by NOS2 inhibition

**Figure 9:**
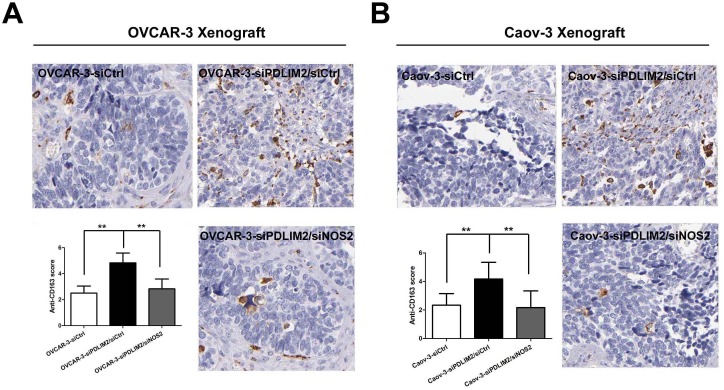
NOS2 inhibition reduces M2 type tumor-associated macrophage infiltration in PDLIM2-low ovarian cancer Representative micro-photographs (left) and quantifications (right) of CD163-positive macrophage content in OVCAR-3 **A.** and Caov-3 **B.** tumor xenografts, respectively. The data presented are the mean±S.D. (error bars). *, *P* < 0.05; **, *P* < 0.01; ***, *P* < 0.001.

To characterize whether PDLIM2 suppression alters the tumor microenvironment, we analyzed the percentage of M2 type tumor macrophage cell infiltration in an ovarian cancer xenograft by immunohistochemistry. Analyses of CD163-immunostained tumor sections revealed increased M2 type tumor-associated macrophage infiltration in siPDLIM2-transfected OVCAR-3 (Figure 9A) and Caov-3 (Figure 9B) ovarian cancer cell specimens compared to control. However, siNOS2 treatment significantly decreased the percentage of CD163-positive cells in both siPDLIM2-transfected OVCAR-3 and Caov-3 ovarian cancer cell xenografts. These data demonstrate PDLIM2 suppression increases M2 type tumor-associated macrophage recruitment in the ovarian cancer tumor microenvironment, attenuated by NOS2 inhibition.

## DISCUSSION

The lack of efficient early detection strategies and limited treatments options contribute to high mortality in advanced stage ovarian cancer patients [2]. Therefore, comprehension of the underlying molecular mechanisms of ovarian cancer pathogenesis is of utmost importance with potential therapeutic value. In the current study, we demonstrate PDLIM2 expression was inhibited in both ovarian high-grade serous carcinoma and in several human ovarian cancer cell lines compared to normal ovarian tissues and human ovarian surface epithelial cells (HOSE). Functional analysis revealed PDLIM2 is epigenetically repressed in ovarian cancer development. PDLIM2 inhibition promoted ovarian cancer growth both *in vivo* and *in vitro* via NOS2-derived nitric oxide signaling, which increased M2 type macrophage recruitment. Together, these results suggest PDLIM2 has an important role in ovarian cancer pathogenesis, and may be a promising therapeutic target in the treatment of ovarian cancer.

PDLIM2 has a PDZ domain (that binds with the actin cy­toskeleton via α-actinin) and a LIM do­main (that has potential to associate with different protein partners). PDLIM2 shuttles between the cytoskeleton and nucleus, and participates in cytoskeletal signaling, cellular polarization, and cellular migration[9]. Recent studies has demonstrated PDLIM2 specifically targets nuclear p65 for polyubiquitination-mediated proteasomal degradation, terminating NF-KB activation [7]. It has been suggested the C-terminal LIM domain of PDLIM2 is required for promoting ubiquitination of nuclear p65, while its N-terminal PDZ domain shuttles nuclear p65 along the nuclear framework into discrete intranuclear compartments for proteasome-mediated degradation [8]. Correspondingly, PDLIM2 knock-out mice are more sensitive to lipopolysaccharide-induced shock due to enhanced p65 activation and subsequently augmented production of inflammatory cytokines [8]. Apart from its involvment in inflammation, its role as a tumor suppressor has also been highlighted. PDLIM2 is repressed in a variety of tumors, including breast [7] and colon cancer [10]. The molecular mechanisms underlying its decreased expression involve promoter methylation. Heretofore, whether PDLIM2 is similarly repressed in ovarian cancer has not been investigated. Our current study demonstrated PDLIM2 is epigenetically repressed in ovarian cancer, and restoration of PDLIM2 significantly impaired ovarian cancer growth, suggesting an important role of PDLIM2 in ovarian cancer development.

Nitric oxide (NO) is an important regulator of a variety of physiological functions, but continuous exposure to moderate-to-high concentrations of NO may lead to pathological processes such as inflammation, apoptosis, and tumorigenesis [11]. iNOS–NO signaling has been reported to be closely correlated with tumorigenesis, but its contribution in ovarian cancer pathogenesis remain unknown. Previous studies have reported homeoprotein DLX4 regulated inducible nitric oxide synthase-mediated angiogenesis in ovarian cancer [12]. Moreover, Caneba et al. demonstrated NO positively orchestrated the Warburg effect (increased glycolysis with reduced mitochondrial activity during aerobic conditions). NO promotes tumor growth and inhibits mitochondrial respiration in OVCA cells, metabolically shifting these cells towards glycolysis to maintain ATP production. Additionally, NO increases TCA cycle flux and glutaminolysis, suggesting NO decreases ROS levels by increasing NADPH and glutathione levels [13]. Our study demonstrated NOS2 expression was increased in PDLIM2-repressed ovarian cancer cells, increasing intracellular NO synthesis in ovarian cancer cells. Via NO synthesis inhibition, we demonstrated PDLIM2-deficient ovarian cancer cells grow in a NO-dependent manner. Taken together, our results support the vital role NO signaling plays in PDLIM2-repressed ovarian cancer cell growth.

Previous investigations have demonstrated macrophage infiltration in the tumor microenvironment favors tumor growth, angiogenesis, inflammation, metastasis/invasion, and immunosuppression through production of various factors promoting tumor progression. These factors serve as the interface between cancer cells and monocytes/macrophages under inflammatory stimuli *in vitro* and *in vivo*. Macrophages supporting aggressively malignant tumors are mostly the M2 type [14]. The number of M2 type tumor-associated macrophages is correlated with prognosis in different cancer types, including ovarian cancer [15]. Our study demonstrated the percentage of M2 type tumor-associated macrophages is significantly increased in PDLIM2-deficient ovarian cancer specimens of nude mice model compared to control, suggesting the likely involvement of M2 type tumor-associated macrophages with PDLIM2-repressed ovarian cancer cell growth.

Taken together, our data demonstrated PDLIM2 is repressed in ovarian cancer cells compared to normal tissue. Functional analysis revealed PDLIM2 is epigenetically repressed by DNA methylation in ovarian cancer, which is conducive to cellular growth both *in vivo* and *in vitro* via NOS2-derived nitric oxide signaling. M2 type macrophages recruitment was increased in the PDLIM2-repressed ovarian cancer tissue tumor microenvironment. Our results support an important role of PDLIM2 in ovarian cancer pathogenesis, which might be a promising therapeutic target in the treatment of ovarian cancer.

## MATERIALS AND METHODS

### Cell line, expression vectors, and reagents

The human ovarian cancer cell lines OVCAR-3, Caov-3, SKOV-3, were obtained from the American Type Culture Collection (ATCC). COV-504 and A2780 were obtained from the European Collection of Cell Cultures (ECACC). All cell lines were cultured per standard protocols. Human ovarian epithelial (HOSE) cells were isolated as previously described [16]. Plasmids encoding full-length human PDLIM2 were purchased from Gene Copoeia (Guangzhou, China). siRNA oligonucleotides with specificity for PDLIM2 and nontargeting control siRNA containing a scrambled sequence were from GenePharma. Nucleoside analogue 5-aza-2′-deoxycytidine (5-aza-dC), 1400W (N-{[3-(aminomethyl)phenyl]methyl}-ethanimidamide, an irreversible NOS2 inhibitor); PTIO (2-phenyl-4,4,5,5-tetramethylimidazoline-1-oxyl 3-oxide, a NO-scavenger), and crystal violet were purchased from Sigma-Aldrich.

### Immunohistochemistry

Paraffin-embedded normal ovary, fallopian tube, and ovarian cancer specimens were obtained from 43 patients, who had undergone surgical resection from 2009 to 2010. Immunohistochemistry was performed utilizing primary antibodies, including mouse anti-PDLIM2 (diluted 1:200; abcam), rabbit anti-NOS2 (diluted 1:200; Santa Cruz Biotechnology), and rabbit anti-CD163 (diluted 1:200; Santa Cruz Biotechnology) as previously described (12). 10 random images per section were captured, and immunohistochemical staining positivity was determined by calculating the percentage of positive cells and immunostain intensity via Image-Pro Plus version 6.0 (Media Cybernetics, Baltimore, MD). Staining intensities ranged 0 to 10. The mean value of staining intensity of 10 captured images was considered each specimen's staining score. All slides were evaluated by two independent pathologists in a double-blinded manner. Any discrepancy between the two evaluators was resolved by re-evaluation and open deliberation until agreement was reached.

### Real-time PCR analysis

Total RNA was obtained with TRIzol reagent. cDNA was generated with SuperScript II reverse transcriptase (Invitrogen). Real-time PCR assays were conducted as previously described. Primer pairs were PDLIM2 (forward 5′-GCCCATCATGGTGACTAAGG, reverse 5′-ATGGCCACGATTATGTCTCC); GAPDH (forward 5′-CGCTCTCTGCTCCTCCTGTT, reverse 5′-CCATGGTGTCTGAGCGATGT); DNMT1 (forward 5′-GGTTCTTCCTCCTGGAGAATGTC, reverse 5′-GGGCCACGCCGTACTG); DNMT3a (forward 5′-GCCTCAATGTTACCCTGGAA; reverse 5′-CAGCAGATGGTGCAGTAGGA); and DNMT3b (forward 5′-CCCATTCGAGTCCTGTCATT, reverse 5′-GGTTCCAACAGCAATGGACT).

### Data set and survival analysis

The Oncomine database provides publicly available data sets concerning cancer gene expression. The Oncomine database was searched to further compare the expression levels of PDLIM2 and NOS2 in normal tissues and ovarian carcinomas. Kaplan Meier survival analysis data of ovarian cancer patients from the Australian Ovarian Cancer Study (AOCS) was collected [17].

### Immunoblot

For immunoblotting, whole cell lysates were prepared as previously described [18]. Proteins from conditioned medium samples were precipitated by mixing 1 ml sample with 5 ml methanol, followed by 1 hour of incubation at −80°C. Samples were pelleted, dried, and subjected to further immunoblotting. All signals were quantified by QuantityOne software (Bio-Rad), and defined as the ratio of target protein to β-actin.

### Cell growth assays

Cells were seeded into 12-well plates at a density of 5,000 cells per well. Cells received 5-aza-dC (5 μmol/L) or vehicle treatment. The drug-containing medium was replenished each day. Cell density was determined by replacing the medium with 2 μmol/L of calcein AM in 1× dissociation solution (Trevigen) at indicated time points. After 1 hour incubation, diesterase activity (in relative fluorescence units) was measured via Tecan Infinite 200 Microplate Reader (excitation wavelength 485 nm and emission wavelength 520 nm).

### Colony formation assay

100 cells were counted and seeded in triplicate in a 6-well plate, and cultured continuously for 14 days. All subsequent clones were stained with crystal violet and counted via light microscopy. Any clusters exceeding 50 cells was considered a clone, and clonogenic formation rate was calculated.

### Bisulfite genomic DNA sequencing

Genomic DNA from 5-aza-dC–treated or mock-treated cells was isolated via PureLink Genomic DNA Purification Kit (Invitrogen) per manufacturer's protocol. Subsequently, genomic DNA aliquots were treated with sodium bisulfite via EZ DNA Methylation-Gold Kit (Zymo Research), followed by PCR to amplify the PDLIM2 promoter by Hot-Start Taq enzyme (Qiagen). Primers were designed to recognize the bisulfite-modified regions (−1084 to −800) of the PDLIM2 promoter (forward 5′-AGAGGAGTTTATATATATTTAGG, reverse 5′-TACCTAACAACCCTCTCTCC). The PCR products were then directly employed for DNA sequencing or subcloned into the SmaI restriction site of pEGFP-N2 (Clontech) for single colony sequencing to determine the methylation status of the CpG dinucleotides within the PDLIM2 promoter.

### Xenograft study

Xenograft experiments were conducted per national guidelines and approved by the Institutional Animal Care and Use Committee of Sichuan University (Chengdu, Sichuan, China), as done previously [19]. Briefly, 2×10^6^ cancer cells were subcutaneously injected in the right flank of 5-7 week old severely combined immunodeficient (SCID) mice. Tumor growth was assessed over time by twice weekly physical examination and palpation. Mice were sacrificed 21 days after inoculation. Tumors were frozen in liquid nitrogen and stored at −75°C for subsequent analyses.

### Nitrite Level Determination by 4,5-Diaminofluorescein

To determine cellular nitrite production, conditioned supernatants or nitrite standards were added to 4,5-diaminofluorescein (DAF-2; Cayman Chemical). Samples were analyzed post-acidification and neutralization for fluorescence (measured with lex=488nm and lem=525 nm). All results were normalized to cellular protein content (measured by Bradford assay; Bio-Rad).

### Statistical analysis

GraphPad Prism 5.0d for Mac (GraphPad Software) was utilized for all statistical analyses. Data was reported as mean±standard deviation. Student's *t* test (two-tailed) assessed the significance of differences between two groups. *p* values<0.05 and 0.01 were respectively considered statistically significant and highly statistically significant. Spearman correlation analyzed the potential correlation between two different experimental parameters (*, *P* < 0.05; **, *P* < 0.01).

## References

[1] Kipps E, Tan DS, Kaye SB (2013). Meeting the challenge of ascites in ovarian cancer: new avenues for therapy and research. Nat Rev Cancer.

[2] Nick AM, Coleman RL, Ramirez PT, Sood AK (2015). A framework for a personalized surgical approach to ovarian cancer. Nat Rev Clin Oncol.

[3] Purdie DM, Bain CJ, Siskind V, Webb PM, Green AC (2003). Ovulation and risk of epithelial ovarian cancer. Int J Cancer.

[4] Ose J, Schock H, Tjønneland A, Hansen L, Overvad K, Dossus L, Clavel-Chapelon F, Baglietto L, Boeing H, Trichopolou A, Benetou V, Lagiou P, Masala G (2015). Inflammatory markers and risk of epithelial ovarian cancer by tumor subtypes: the EPIC cohort. Cancer Epidemiol Biomarkers Prev.

[5] Haria D, Trinh BQ, Ko SY, Barengo N, Liu J, Naora H (2015). The homeoprotein DLX4 stimulates NF-kappa B activation and CD44-mediated tumor-mesothelial cell interactions in ovarian cancer. Am J Pathol.

[6] Hsu S, Kim M, Hernandez L, Grajales V, Noonan A, Anver M, Davidson B, Annunziata CM (2012). IKK-epsilon coordinates invasion and metastasis of ovarian cancer. Cancer Res.

[7] Qu Z, Fu J, Yan P, Hu J, Cheng SY, Xiao G (2010). Epigenetic repression of PDZ-LIM domain-containing protein 2: implications for the biology and treatment of breast cancer. J Biol Chem.

[8] Tanaka T, Grusby MJ, Kaisho T (2007). PDLIM2-mediated termination of transcription factor NF-kappaB activation by intranuclear sequestration and degradation of the p65 subunit. Nat Immunol.

[9] Bowe RA, Cox OT, Ayllon V, Tresse E, Healy NC, Edmunds SJ, Huigsloot M, O'Connor R (2014). PDLIM2 regulates transcription factor activity in epithelial-to-mesenchymal transition via the COP9 signalosome. Mol Biol Cell.

[10] Qu Z, Yan P, Fu J, Jiang J, Grusby MJ, Smithgall TE, Xiao G (2010). DNA methylation-dependent repression of PDZ-LIM domain-containing protein 2 in colon cancer and its role as a potential therapeutic target. Cancer Res.

[11] Fukumura D, Kashiwagi S, Jain RK (2006). The role of nitric oxide in tumour progression. Nat Rev Cancer.

[12] Trinh B, Ko SY, Haria D, Barengo N, Naora H (2015). The homeoprotein DLX4 controls inducible nitric oxide synthase-mediated angiogenesis in ovarian cancer. Mol Cancer.

[13] Caneba CA, Yang L, Baddour J, Curtis R, Win J, Hartig S, Marini J, Nagrath D (2014). Nitric oxide is a positive regulator of the Warburg effect in ovarian cancer cells. Cell Death Dis.

[14] Mills CD (2015). Anatomy of a discovery: m1 and m2 macrophages. Front Immunol.

[15] Zhang QW, Liu L, Gong CY, Shi HS, Zeng YH, Wang XZ, Zhao YW, Wei YQ (2012). Prognostic significance of tumor-associated macrophages in solid tumor: a meta-analysis of the literature. PloS One.

[16] Bitler BG, Nicodemus JP, Li H, Cai Q, Wu H, Hua X, Li T, Birrer MJ, Godwin AK, Cairns P, Zhang R (2011). Wnt5a suppresses epithelial ovarian cancer by promoting cellular senescence. Cancer Res.

[17] Gyorffy B, Lanczky A, Szallasi Z (2012). Implementing an online tool for genome-wide validation of survival-associated biomarkers in ovarian-cancer using microarray data from 1287 patients. Endocr Relat Cancer.

[18] Zhou S, Yi T, Liu R, Bian C, Qi X, He X, Wang K, Li J, Zhao X, Huang C, Wei Y (2012). Proteomics identification of annexin A2 as a key mediator in the metastasis and proangiogenesis of endometrial cells in human adenomyosis. Mol Cell Proteomics.

[19] Zhang X, Jiang F, Li P, Li C, Ma Q, Nicosia SV, Bai W (2005). Growth suppression of ovarian cancer xenografts in nude mice by vitamin D analogue EB1089. Clin Cancer Res.

